# Grand Rapids Veterinary College

**Published:** 1899-05

**Authors:** 


### GRAND RAPIDS VETERINARY COLLEGE.

The second annual commencement exercises of this college took place Thursday evening, March 23d, at the College Auditorium. The building was crowded long before the exercises opened. Dr. Herrick, acting as the president of the Board of Trustees, presented the graduating class, numbering five, with their diplomas, as follows: L. M. Newman, James Ackerson, Chelsea, Mich.; John Krauf, R. S. Coleman, Sparta, and W. A. Seabury, Paw Paw, Mich. The exercises were of considerable length, and included two papers by the graduating class. Perhaps the most unique and interesting feature of the programme was the examination of little Opal Conkey, on the subject of osteology. The memory which the little child displayed was marvellous, and called forth an ovation from the audience. Drs. Griswold, Fuller, and Herrick were present, and all but Dr. Fuller made short addresses to the class and their friends.

